# Temporal Trends in and Associations With Nonsteroidal Anti‐inflammatory Drug Prescription in Adult and Pediatric Patients With Inflammatory Bowel Disease

**DOI:** 10.1002/acr.25650

**Published:** 2025-12-17

**Authors:** Adam S. Mayer, Rui Xiao, Andrew Grossman, Meenakshi Bewtra, Michael D. George, Pamela F. Weiss

**Affiliations:** ^1^ University of Pennsylvania Philadelphia; ^2^ Children's Hospital of Philadelphia Philadelphia Pennsylvania

## Abstract

**Objective:**

Recent inflammatory bowel disease (IBD) treatment guidelines have recommended against nonsteroidal anti‐inflammatory drug (NSAID) use despite prevalent musculoskeletal symptoms and opioid overuse in this population. Given the discordance between changing national guidelines and potential clinical utility, we sought to assess national temporal trends in prescription NSAID and opioid use for patients with IBD and factors associated with NSAID fill trends.

**Methods:**

This retrospective cohort study of adult and pediatric IBD patients used administrative claims data from 2000 to 2022. Prescription NSAID and opioid fills per calendar year were assessed. Wilcoxon‐Cuzick test of trend and generalized estimating equation models evaluated NSAID and opioid fill trends and assessed characteristics associated with NSAID use.

**Results:**

Among the 361,025 IBD patients, there was a significant decreasing trend in the proportion prescribed NSAIDs over time (*P* < 0.01). Fill rates of NSAIDs were markedly lower than opioids across the study period despite an increase in musculoskeletal pain codes. In the multivariable model, opioid prescription (odds ratio [OR] 2.13, 95% confidence interval [CI] 2.11–2.15), a diagnostic code for osteoarthritis (OR 1.57, 95% CI 1.55–1.59), or unspecified joint pain (OR 1.54, 95% CI 1.52–1.56) had strong independent associations with NSAID fill, whereas an age <18 or ≥80 years were associated with significantly lower odds of NSAID fill.

**Conclusion:**

NSAIDs are used by a minority of patients with IBD, with decreasing prescription rates over time despite high rates of opioid use and a doubling of musculoskeletal complaints. NSAID safety needs more thorough examination as an effective and potentially lower‐risk analgesic option for patients of all ages with IBD.

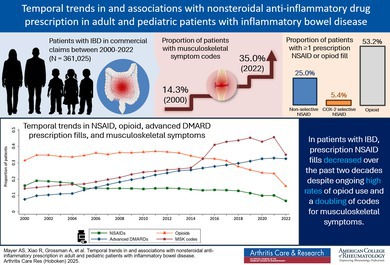

## INTRODUCTION

Acute and chronic pain are prominent features of inflammatory bowel disease (IBD), which can significantly impact quality of life.^1^ The etiology of pain in patients with IBD may be multifactorial but includes inflammatory arthritis or enthesitis in up to 39% of patients.^2^ Because of their ease of access, low cost, and effectiveness for a myriad of conditions, including arthritis, nonsteroidal anti‐inflammatory drugs (NSAIDs) are an attractive therapeutic option for patients with IBD and musculoskeletal complaints.

**SIGNIFICANCE & INNOVATIONS**

- In a large United States cohort of adult and pediatric patients with IBD, we found an overall decreasing trend in prescription NSAID fills over the last two decades despite ongoing high rates of opioid prescription across age groups and a doubling of codes for musculoskeletal pain.
- Several factors independently associated with prescription NSAID use were identified, including codes for osteoarthritis and unspecified joint pain, suggesting that musculoskeletal symptoms are a key driver of NSAID use in patients with IBD.
- Our findings highlight the need to better understand the true risk of NSAIDs for patients with IBD as an effective and potentially lower‐risk analgesic option.

However, there is concern that NSAIDs may precipitate intestinal inflammation in patients with IBD. A large 2016 cohort study of patients with self‐reported IBD in remission found an increased risk of relapse (risk ratio [RR] 1.65, 95% confidence interval [CI] 1.12–2.44) in those with Crohn disease (CD) subtype who reported at least five NSAID doses per month compared to those with lower intake, although an increased risk associated with acetaminophen exposure was similarly identified.^3^ In contrast, a recent systematic review and meta‐analysis found no significant increase in risk of IBD exacerbation associated with NSAID exposure (pooled RR 1.29, 95% CI 0.92–1.80). Despite mixed data, the most recent 2018 and 2019 American College of Gastroenterology (ACG) treatment guidelines for both CD and ulcerative colitis (UC) strongly recommend against the use of NSAIDs, given the possible risk of intestinal flare.^4^, ^5^

Although the mixed evidence poses a challenge for clinicians caring for adult patients with IBD, even greater challenges exist in the care of pediatric patients with IBD because there are no large studies assessing NSAID risk in this population. Furthermore, the decision to use NSAIDs in patients with IBD is counterweighted by more toxic alternatives including opioids, glucocorticoids, and escalating background immunosuppressive therapy. It is not clear how NSAID prescribing patterns for those with IBD have changed longitudinally in light of sparse and often conflicting evidence and changing national guidelines nor how these trends compare with those of opioids, which are a major alternative analgesic therapy in this population. As such, we sought to evaluate temporal trends in prescription NSAID and opioid fills over time in both adult and pediatric patients with IBD as well as assess factors associated with prescription NSAID use.

## MATERIALS AND METHODS

The protocol for the conduct of this study was reviewed by the Children's Hospital of Philadelphia Institutional Review Board and determined to be not human subject research.

### Data source

This retrospective cohort study used data from January 2000 to June 2022 in Optum's deidentified Clinformatics Data Mart Database (CDM), a national database of administrative health claims for adult and pediatric members of commercial and Medicare Advantage health plans. Available data included deidentified patient characteristics, International Classification of Diseases (ICD)‐9 and ICD‐10 diagnosis codes, National Drug Codes (NDCs) for medications, prescription fill dates, pill quantity, and days supplied for a given prescription.

### Patient population

Adult (age ≥ 18 years) and pediatric (age < 18 years) patients with IBD were included. A diagnosis of IBD was defined based on validated algorithms^6^, ^7^, ^8^, ^9^, ^10^, ^11^ and required all of the following: (1) at least two inpatient or outpatient diagnosis codes of CD (ICD‐9: 556.xx / ICD‐10: K51.xx) and/or UC (ICD‐9: 555.xx / ICD‐10: K50.xx) at least 30 days apart; (2) at least one diagnosis code had to be from an outpatient encounter; and (3) a minimum of six months of continuous enrollment. Select prior claims database studies in pediatric IBD have included less specific codes for indeterminate and noninfective colitis,^12^ which we excluded to increase the specificity of our IBD definition and also allow more accurate comparison between the adult and pediatric cohorts by using identical codes for inclusion in both groups. Patients under six years old at the time of meeting IBD criteria were excluded because of the possibility of very‐early–onset IBD in this population, a distinct clinical entity from traditional IBD.^13^ Patients without pharmacy claims data were also excluded.

The index date was defined as the date the patient met the IBD criteria. All data for outcome analysis started after the index date.

### Exposure

The primary exposure was calendar year. Data from each calendar year for a given patient were included if there was at least six months of enrollment data available for that year. Each included calendar year was considered as a separate observation for every patient. Person‐months of exposure per calendar year were adjusted for in the primary analysis to account for differential enrollment periods.

### Outcomes

The primary outcome was the proportion of patients with more than or equal to one oral NSAID prescription fill each calendar year. Prescription fills for both nonselective and cyclooxygenase‐2 (COX‐2) selective NSAIDs were assessed (**Supplemental Table** 1). NSAID receipt was dichotomized as ever or never (more than or equal to one NSAID prescription fill) to characterize the overall cohort. NSAID fills by calendar year were used to assess trends in prescription NSAID use over time. A secondary outcome was the proportion of patients with more than or equal to one opioid fill in each calendar year. A sensitivity analysis assessed longitudinal trends of chronic NSAID or opioid use, defined as having at least two NSAID or opioid fills in a calendar year.

### Covariates

Potential factors associated with prescription NSAID use included age at the start of calendar year, sex, United States (US) census division (Northeast, South, Midwest, and West), IBD type (CD, UC, or indeterminate colitis [IC]), opioid exposure (or NSAID exposure when assessing opioid fill secondary outcome), nonglucocorticoid immunosuppression regimen (none, conventional synthetic disease‐modifying antirheumatic drugs [csDMARD], biologic DMARDs [bDMARDs], or targeted synthetic DMARDs [tsDMARDs]), arthritis, chronic musculoskeletal pain, depression, anxiety, psychotic disorders, alcohol use, substance use, nicotine/tobacco use, and months enrolled in CDM during eligible calendar years. These covariates were a priori chosen given prior publications demonstrating their association with opioid use and disease severity in adult and pediatric IBD,^12^, ^14^, ^15^, ^16^ which are likely proxies for NSAID exposure. For IBD type, a patient was assigned a diagnosis of CD or UC if ≥75% of IBD codes during the study period were CD‐ or UC‐specific codes, respectively. A patient was labeled as IC if they did not meet this majority threshold for CD or UC codes. Immunosuppression prescription was determined by corresponding NDC or health care common procedure coding system codes. tsDMARDs (ie, Janus kinase inhibitors) were combined with bDMARDs into a category of advanced DMARD therapies because tsDMARDs were first introduced to the US market more than halfway through the study period and used by only a small minority of patients in this study. Arthritis, chronic musculoskeletal pain, mental health disorders, alcohol, substance and nicotine/tobacco use were determined by the presence of more than or equal to corresponding ICD‐9 or ICD‐10 code (**Supplemental Table** 2) in a given calendar year.

### Statistical analysis

Differences in characteristics between (1) adult and pediatric patients with IBD, (2) adult and pediatric patients with IBD and more than or equal to NSAID fill versus none, and (3) those with more than or equal to one NSAID fill during the study period vs those without were summarized using descriptive statistics and compared using Pearson's chi‐squared test or *t*‐test as appropriate. The Wilcoxon‐Cuzick test of trend was used to assess the overall trend in NSAID prescription over time. Generalized estimating equations (GEEs) were used to further determine when adjusting for covariates how the proportion of patients receiving more than or equal to prescription NSAID or more than or equal to opioid during a calendar year changed over time. The GEE model accounted for within‐subject correlation as individuals could contribute data for more than one calendar year with time‐updating covariates each calendar year. The GEE model was stratified by five age groups (ages 6–17, 18–39, 40–59, 60–79, and 80–99 years). Covariates were included in the multivariable GEE model if there was a significant (*P* < 0.05) association with the primary outcome in univariable models. Linear splines in the GEE models with knots prespecified at calendar years of 2004 and 2018 were used to capture the nonlinear temporal tend. These knots were chosen because of (1) the Federal Drug Administration (FDA) withdrawal of the COX‐2 selective inhibitor rofecoxib from the US market in late 2004 and (2) the 2018 ACG (ACG) CD guidelines being the first full‐length IBD guidelines to strongly recommend against NSAID use. The slopes of each segment were tested and compared using postestimation commands in Stata. For analysis of opioid fills, a spline regression model was built using a knot at 2012 based on the visual trend. Given that arthritis and chronic musculoskeletal pain are common indications for NSAID prescription in patients with IBD,^17^, ^18^ temporal trends of these diagnostic codes were assessed. Finally, because NSAID and opioid fills may be influenced by changing use of advanced DMARD therapies over time, trends in advanced DMARD fills were similarly evaluated. Given the expected sample size of >300,000 patients, there was no anticipation of power limitations for the primary analysis. All statistical analyses were performed using Stata version 18.0.

## RESULTS

### Cohort characteristics

**Figure 1:**
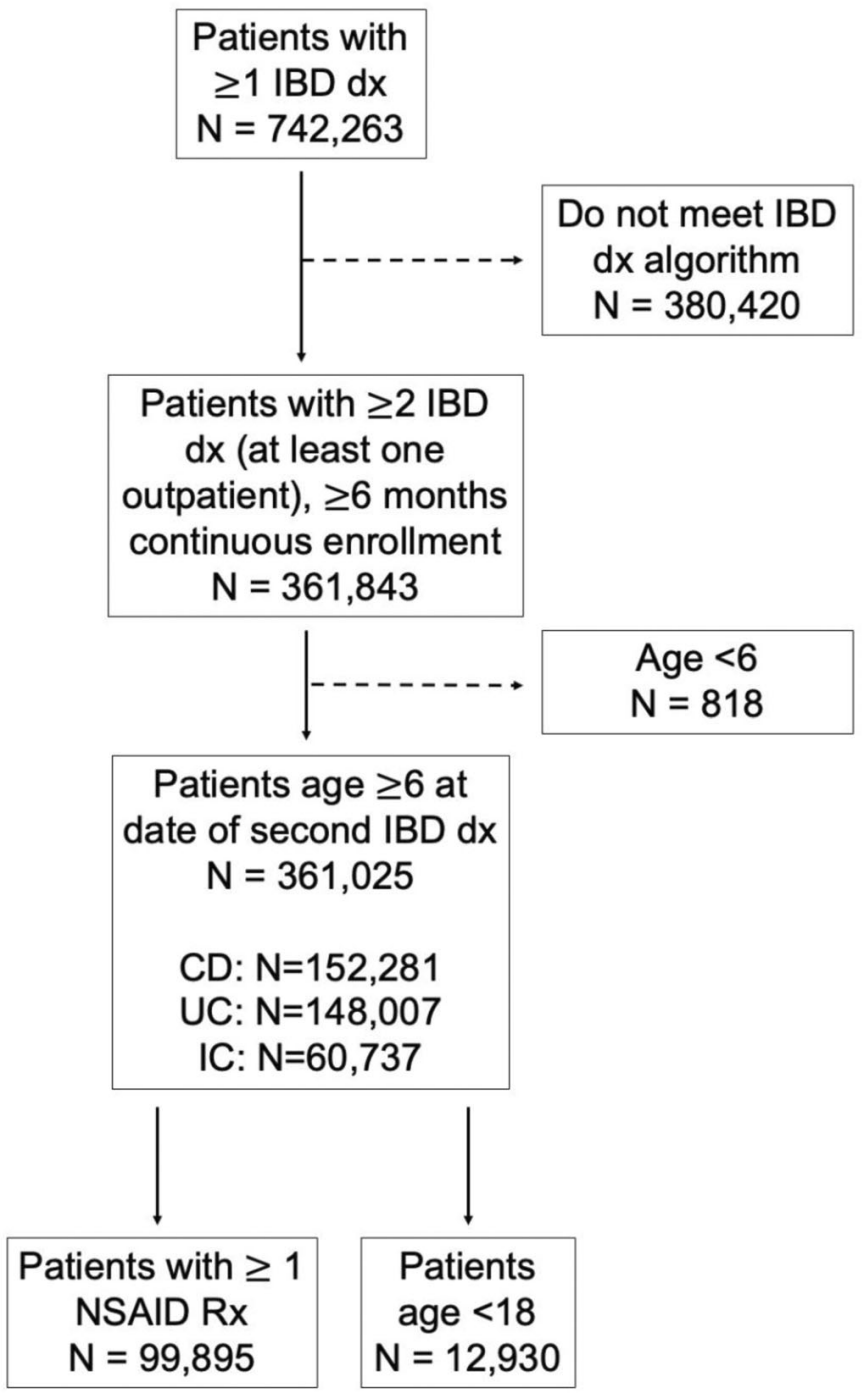
Flow diagram of study cohort. Dashed lines represent excluded patients. CD, Crohn disease; dx, diagnosis; IBD, inflammatory bowel disease; IC, indeterminate colitis; NSAID, nonsteroidal anti‐inflammatory drug; Rx, prescription; UC, ulcerative colitis.

A flow diagram is shown in Figure 1. A total of 361,025 patients met inclusion criteria, including 12,930 (3.6%) patients <18 years old. The cohort characteristics stratified by age group are displayed in **Table** 1. Compared to those <18 years of age, adults with IBD were more likely to be female (*P* < 0.01), have more than or equal to one NSAID fill during the study period (28.5% vs 7.6%; *P* < 0.01) including for nonselective (*P* < 0.01) and COX‐2 selective NSAIDs (*P* < 0.01), or have more than or equal to one opioid fill during the study period (54.3% vs 23.5%; *P* < 0.01). Adults were significantly less likely to receive csDMARDs (*P* < 0.01) or bDMARDs/tsDMARDs (*P* < 0.01) than children. Adult patients had a significantly higher prevalence of several comorbidities including inflammatory arthritis (specifically, rheumatoid arthritis and spondylarthritis codes), osteoarthritis, other joint pain not otherwise specified (NOS), and chronic musculoskeletal pain (*P* < 0.01 for all). Similar differences between age groups were followed when assessing the subgroup of those who received more than or equal to one NSAID prescription during the study period, with the exception of spondyloarthritis codes, which did not significantly differ between adult and pediatric patients (13.5% vs 12.8%; *P* = 0.34).

**Table 1:** Characteristics of patients enrolled, stratified by age group*

	All (n = 361,025)	Age ≥18 years (n = 348,095)	Age <18 years (n = 12,930)
Demographics			
Years enrolled, median (IQR)	3 (2–5)	3 (2–5)	3 (2–5)
Months enrolled per calendar year, median (IQR)	12 (10.5–12)	12 (10.5–12)	12 (10.5–12)
Age at index date, median (IQR)	50 (34–65)	50 (35–65)	14 (12–16)
Female sex, n (%)	197,737 (54.8)	191,982 (55.2)	5,755 (44.5)
Geographic region, n (%)			
Northeast	48,744 (13.5)	47,008 (13.5)	1,736 (13.4)
South	147,837 (40.9)	142,652 (41.0)	5,185 (40.1)
Midwest	96,140 (26.6)	92,268 (26.5)	3,872 (29.9)
West	67,909 (18.8)	65,789 (18.9)	2,120 (16.4)
Unknown	395 (0.1)	378 (0.1)	17 (0.1)
Clinical features, n (%)			
IBD type			
Crohn's disease	152,281 (42.2)	146,892 (42.2)	5,389 (41.7)
Ulcerative colitis	148,007 (41.0)	142,745 (41.0)	5,262 (40.7)
Indeterminate colitis	60,737 (16.8)	58,458 (16.8)	2,279 (17.6)
Inflammatory arthritis	42,848 (11.9)	42,127 (12.1)	721 (5.6)
Rheumatoid arthritis	22,493 (6.2)	22,153 (6.4)	340 (2.6)
Spondyloarthritis	27,352 (7.6)	26,836 (7.7)	516 (4.0)
JIA	392 (0.1)	229 (0.1)	163 (1.3)
Osteoarthritis	113,128 (31.3)	112,942 (31.9)	186 (1.6)
Other joint pain NOS	120,580 (33.4)	119,071 (33.6)	1,509 (12.6)
Chronic musculoskeletal pain	75,082 (20.8)	74,243 (20.9)	839 (7.0)
Depression	108,138 (30.0)	106,328 (30.0)	1,810 (15.2)
Anxiety	111,687 (30.9)	109,596 (30.9)	2,091 (17.5)
Psychotic disorder	20,928 (5.8)	20,676 (5.8)	252 (2.1)
Alcohol use disorder	14,014 (3.9)	13,948 (3.9)	66 (0.6)
Substance use	44,298 (12.3)	44,085 (12.4)	213 (1.8)
Nicotine/tobacco use	95,485 (26.4)	95,365 (26.9)	90 (0.8)
Medications, n (%)			
≥1 NSAID fill	100,084 (27.7)	99,099 (28.5)	985 (7.6)
≥1 COX‐2 NSAID fill	19,359 (5.4)	19,189 (5.5)	170 (1.3)
≥1 nonselective NSAID fill	90,316 (25.0)	89,452 (25.7)	864 (6.7)
≥1 opioid fill	192,093 (53.2)	189,056 (54.3)	3,037 (23.5)
Immunosuppression			
None	108,158 (30.0)	106,939 (30.7)	1,219 (9.4)
csDMARD	218,183 (60.4)	209,135 (60.1)	9,048 (70.0)
bDMARD/tsDMARD	107,210 (29.7)	101,876 (29.3)	5,334 (41.3)

* bDMARD, biologic disease‐modifying antirheumatic drug; COX‐2, cyclooxygenase‐2; csDMARD, conventional synthetic DMARD; IBD, inflammatory bowel disease; IQR, interquartile range; JIA, juvenile idiopathic arthritis; NOS, not otherwise specified; NSAID: nonsteroidal anti‐inflammatory drug, tsDMARD: targeted DMARD.

A total of 100,084 (27.7%) patients had at least one prescription NSAID fill during the study period, including 19,359 (5.4%) with at least one COX‐2 selective NSAID fill. Of those patients with at least one NSAID fill, the median number of fills was two (interquartile range [IQR] 1–5) over a median of five years of follow‐up, the median number of pills supplied per prescription was 66 (IQR 30–210), and the median number of prescription days supplied was 35 (IQR 15–127). The NSAIDs most commonly prescribed included meloxicam (26.4%), celecoxib (18.8%), ibuprofen (15.5%), naproxen (11.8%), and diclofenac (10.1%) (**Supplemental Figure** 1). A total of 192,093 patients (53.2%) had at least one opioid prescription filled. Of those with more than or equal to one opioid fill, the median number of fills was three (IQR 1–10).

### Factors associated with prescription NSAID use

In the multivariable model, several variables had a significant and independent association with NSAID prescription (**Table** 2). Patients with an opioid prescription in the same year or diagnoses of inflammatory arthritis, osteoarthritis, or other joint pain NOS were significantly more likely to use NSAIDs. Other clinical characteristics, including diagnoses of chronic musculoskeletal pain, depression, anxiety, or psychotic disorder, had smaller significant effect estimates, whereas IBD type, alcohol use, substance use, and nicotine/tobacco use were not significantly associated with prescription NSAID use. Conversely, male sex, age <18 years or age ≥80 years, and use of advanced immunosuppression (bDMARDs/tsDMARDs alone or in combination with csDMARDs) were independently associated with a lower odds of NSAID prescription. The number of eligible months per calendar year was weakly associated with NSAID prescription.

**Table 2:** Results of univariable and multivariable GEE regression models for factors associated with NSAID prescription*

Factor	Univariate OR	95% CI	Multivariate OR	95% CI
Demographics				
Age, years, (reference: 18, 19, 20, 21, 22, 23, 24, 25, 26, 27, 28, 29, 30, 31, 32, 33, 34, 35, 36, 37, 38, 39)				
6–17	0.49	0.46–0.52	0.54	0.51–0.58
40–59	1.39	1.36–1.41	1.19	1.18–1.21
60–79	1.25	1.23–1.27	1.06	1.04–1.08
80–99	0.73	0.71–0.75	0.67	0.65–0.69
Male sex	0.76	0.75–0.77	0.89	0.88–0.90
Calendar year	0.85	0.85–0.86	0.96	0.96–0.96
Number of eligible months per calendar year	1.09	1.09–1.09	1.04	1.04–1.04
Years enrolled in CDM	1.00	1.00–1.00	0.99	0.99–1.00
Geographic region (reference: northeast)				
South	1.29	1.27–1.32	1.12	1.10–1.14
Midwest	1.00	0.97–1.02	0.91	0.89–0.93
West	0.93	0.90–0.95	0.88	0.71–1.13
Clinical features				
IBD type (reference: CD)				
UC	1.01	1.00–1.02	1.00	0.99–1.02
IC	1.03	1.01–1.05	0.99	0.98–1.01
≥1 Opioid fill in same year	2.64	2.62–2.67	2.13	2.11–2.15
Immunosuppression (reference: none)				
csDMARD	1.01	1.00–1.03	1.00	0.99–1.01
bDMARD/tsDMARD	0.84	0.82–0.86	0.88	0.87–0.89
csDMARD+bDMARD/tsDMARD	0.92	0.91–0.94	0.90	0.88–0.91
Inflammatory arthritis	1.83	1.80–1.86	1.25	1.23–1.28
Osteoarthritis	2.10	2.08–2.12	1.57	1.55–1.59
Other joint pain NOS	1.63	1.61–1.65	1.54	1.52–1.56
Chronic musculoskeletal pain	1.67	1.65–1.69	1.11	1.10–1.12
Depression	1.32	1.31–1.34	1.03	1.02–1.04
Anxiety	1.27	1.26–1.29	1.04	1.02–1.06
Psychotic disorders	1.44	1.41–1.48	1.10	1.07–1.13
Substance use	1.33	1.31–1.36	1.01	0.98–1.02
Alcohol use	1.22	1.18–1.27	1.01	0.98–1.05
Nicotine/tobacco use	1.31	1.29–1.32	1.00	0.99–1.02

* bDMARD, biologic disease‐modifying antirheumatic drug; CD, Crohn disease; CDM, Clinformatics Data Mart Database; CI, confidence interval; csDMARD, conventional synthetic DMARD; GEE, generalized estimating equation; IBD, inflammatory bowel disease; IC, indeterminate colitis; NOS, not otherwise specified; NSAID, nonsteroidal anti‐inflammatory drug; OR, odds ratio; tsDMARD, targeted synthetic DMARD; UC, ulcerative colitis.

### Temporal trends in prescription NSAID and opioid use

**Figure 2:**
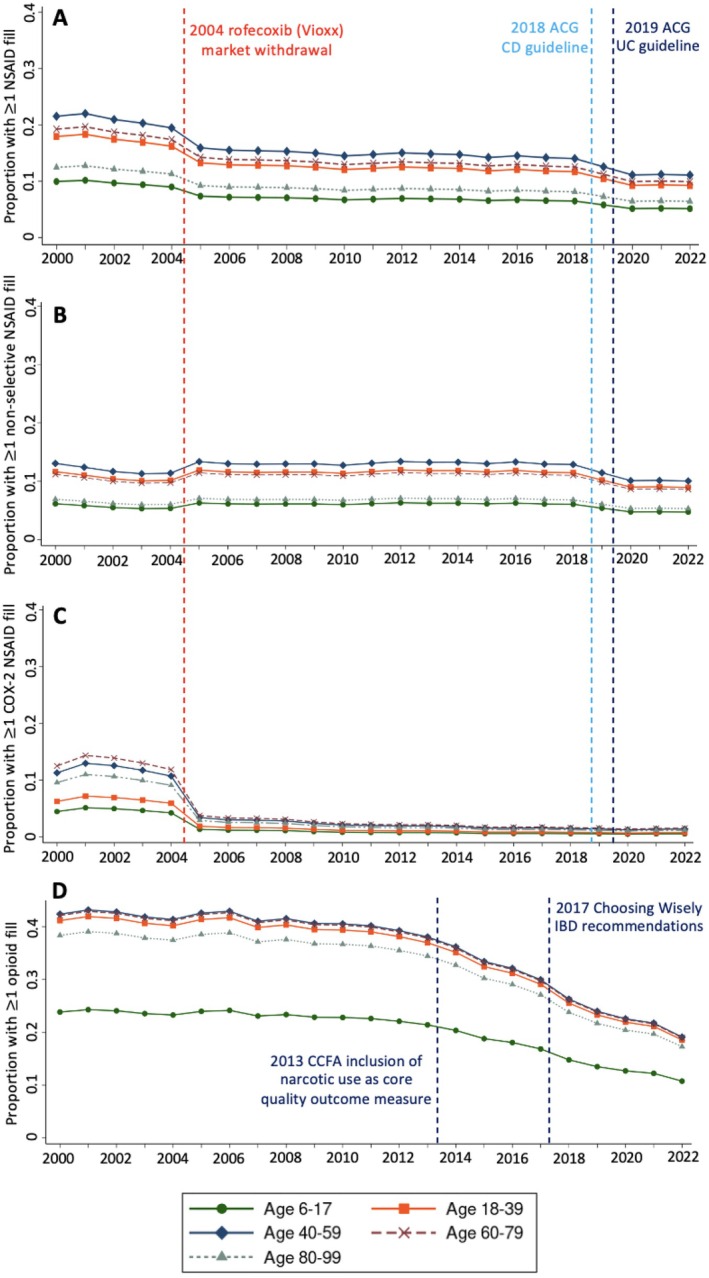
Longitudinal trends in (A) prescription NSAID, (B) nonselective NSAID, (C) COX‐2 selective NSAID, and (D) opioid fills. Annual proportions of prescriptions fills are estimated from the generalized estimating equation model adjusted for covariates. Relevant gastroenterological guidelines advising against NSAID use in IBD are indicated by the vertical dashed lines in (A)–(C). Relevant national public health and quality of care guidelines for opioid use in IBD are indicated by the vertical dashed lines in (D). ACG, American College of Gastroenterology; AGA, American Gastroenterological Association; CCFA, Crohn's & Colitis Foundation of America; CD, Crohn disease; COX‐2, cyclooxygenase‐2; IBD, inflammatory bowel disease; NSAID, nonsteroidal anti‐inflammatory drug; UC, ulcerative colitis.

Trends in NSAID prescriptions by calendar year are illustrated in Figure 2A–C. There was a significant decrease in the proportion of patients receiving NSAIDs over time (*P* < 0.01), from 16.0% in 2000 to 6.7% in 2022. There were significant changes in NSAID prescriptions at 2004 and 2018 in the linear spline regression model (*P* < 0.01 for each). Similar spline regressions for the NSAID subtypes revealed a significant decreasing trend in COX‐2 selective NSAID fills after 2004 and nonselective NSAID fills after 2018 (*P* < 0.01 for both). These trends were consistent across age groups.

**Figure 3:**
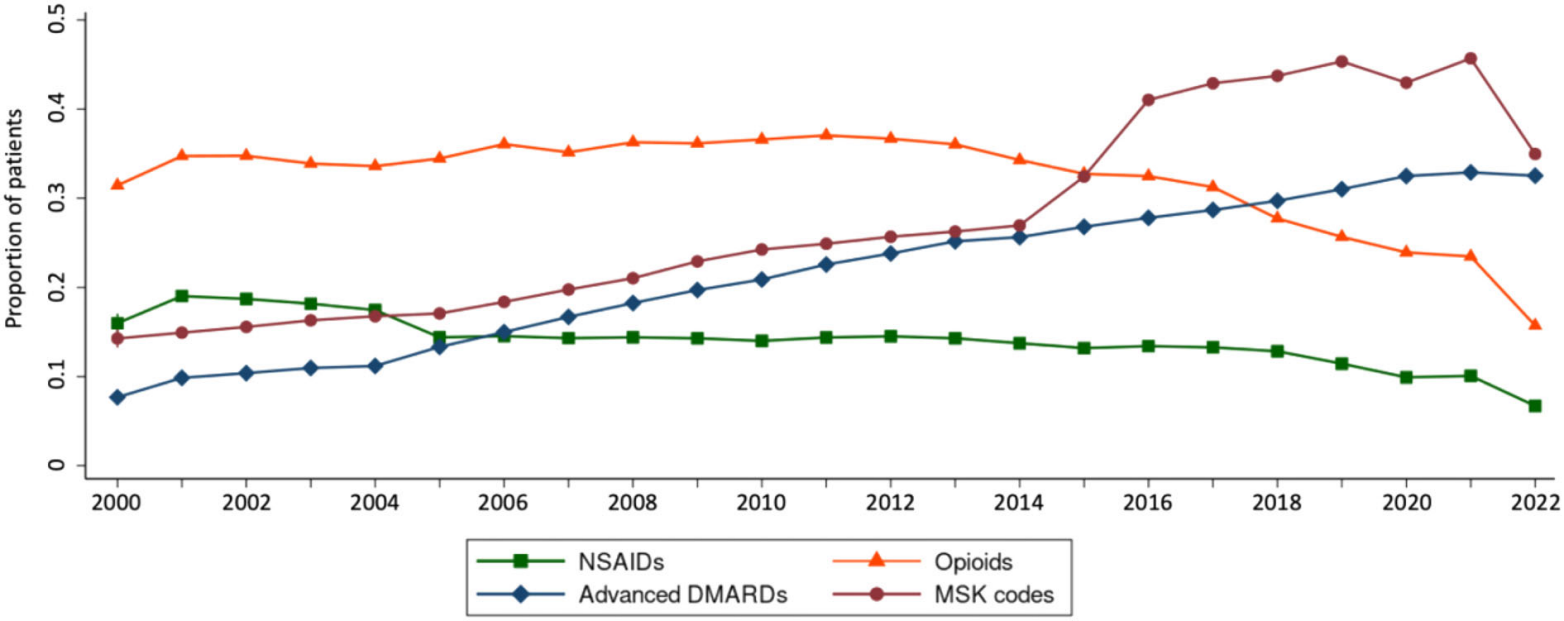
Comparative trends in musculoskeletal symptom codes and prescription NSAID, opioid, and advanced DMARD (includes biologic and targeted synthetic DMARDs) fills. MSK codes refer to diagnosis codes for arthritis or chronic musculoskeletal pain. DMARD: disease modifying antirheumatic drug; MSK: musculoskeletal; NSAID, nonsteroidal anti‐inflammatory drug. Color figure can be viewed in the online issue, which is available at http://onlinelibrary.wiley.com/doi/10.1002/acr.25650/abstract.

There was a significant downward trend in the proportion of patients with IBD receiving more than or equal to one opioid prescription over time (Figure 2D; *P* < 0.01). The proportion of patients receiving opioid prescriptions was relatively stable before 2012 (*P* = 0.89), but there was a significant downslope after 2012 (*P* < 0.01), with 15.9% of enrolled patients prescribed opioids in 2022. Despite this downward trend, opioid use remained nearly twice as common as NSAID use across all age groups throughout the study period. In contrast to the decreasing trend in NSAID and opioid use over time, the proportion of patients with advanced DMARD fills and codes for arthritis or chronic musculoskeletal pain in a given calendar year significantly increased over time (*P* < 0.01), with musculoskeletal symptom codes increasing from 14.3% in 2000 to 35.0% in 2022 (Figure 3). The longitudinal pattern of NSAID and opioid fills in the primary analysis did not differ across IBD subtypes or among chronic users (**Supplemental Figures** 2 and 3).

## DISCUSSION

Leveraging a large cohort of adult and pediatric patients with IBD from a national health care administrative claims database, we found an overall decreasing trend in the proportion of patients receiving prescription NSAIDs over time in all age groups, including (1) a marked sustained decrease in COX‐2 selective inhibitors after 2004, when rofecoxib was withdrawn from the US market, and (2) a further decrease in all prescription NSAID fills after the most recent ACG IBD treatment guideline recommendations. The proportion of patients receiving prescription NSAIDs remained well below that of those receiving opioids across all calendar years irrespective of age group, despite a more‐than‐doubling of codes for musculoskeletal pain over time. Several factors strongly associated with NSAID use were identified including opioid prescription or codes for inflammatory arthritis, osteoarthritis, or joint pain NOS in the same calendar year. Pediatric patients had the lowest odds of NSAID prescription. There are several strengths to our study. The inclusion of a pediatric cohort is novel because to the best of our knowledge there are no published studies assessing trends in NSAID prescription in pediatric IBD and particularly how this directly compares to adult cohorts. Our study spanned 22 years and thus allowed for the determination of longer‐term comparative trends in NSAID and opioid fills across changing guidelines and a dynamic landscape of novel therapies for the treatment of IBD and ultimately highlight the need for further study of NSAID safety as an analgesic that may be less toxic than opioids in this population.

The decreasing trend in prescription NSAID use over time in our US‐based cohort is consistent with that reported previously in Danish patients with IBD^19^ as well as longitudinal NSAID prescription trends in other diseases such as axial spondyloarthritis and cardiovascular disease (CVD).^20^, ^21^, ^22^ The precipitous drop in COX‐2 prescription fills after the FDA withdrawal of rofecoxib in 2004 is a well‐established phenomenon and there appears to have been a contemporaneous compensatory increase in nonselective NSAID prescriptions, which has been previously demonstrated outside of IBD.^23^ Subsequent to 2004, the proportion of patients using COX‐2 selective NSAIDs remained markedly lower than nonselective NSAIDs, despite data suggesting relative safety in patients with IBD.^24^ However, there was also a significant decrease in the proportion of patients with nonselective NSAID prescriptions after 2018. We hypothesize this may be due to the 2018 and 2019 ACG guidelines^4^, ^5^ strongly recommending against the use of NSAIDs in patients with IBD, which were the first full‐length IBD guidelines to do so and thus highlighting the impact of national treatment guidelines on provider prescribing practices. As supported by the multivariable model, the advent of more effective immunosuppressive medications over time (eg, bDMARDs and tsDMARDs) is another factor likely contributing to the decreasing rate of prescription NSAID use due to better control of underlying IBD and associated musculoskeletal disease, but there is still a decreasing trend in NSAID prescription independent of immunosuppressive regimen. More recent treatment protocols emphasizing earlier treatment of IBD with biologic and small molecule therapies may also contribute to this trend. The pattern of NSAID use over time in children with IBD mirrored that of adult patients but with a lower proportion of patients with fills each year for each NSAID subtype and with a notably low prevalence of COX‐2 selective NSAID use overall. The lower proportion of NSAID prescriptions may be related to the earlier and more frequent use of biologic therapies observed in the pediatric group, which may result in lower disease activity, as well as the lower prevalence of comorbidities relative to adults, including arthritis and chronic musculoskeletal pain.^25^

The proportion of patients receiving opioids decreased over time after 2012, consistent with several prior studies in adult and pediatric IBD.^12^, ^26^, ^27^, ^28^ This trend may be due in large part to national shifts in medical practice and increased awareness of risks associated with opioid use.^29^, ^30^, ^31^ These shifts in practice are also reflected in the inclusion of narcotic medication use as a core outcome measure in the quality indicators for IBD from the Crohn's & Colitis Foundation of America in 2013^32^ as well as the 2017 recommendation to avoid long‐term opioid therapy for abdominal pain in IBD from the Choosing Wisely campaign.^33^ Nevertheless, opioid fills were nearly twice as common as NSAID fills in our cohort, and the prevalence of opioid fills remained markedly higher than that of NSAIDs across the entire 22 year study period, acknowledging that data on over‐the‐counter NSAID use are missing in claims databases, whereas opioids are only obtained by prescription. These findings are consistent with a large 2014 Canadian retrospective cohort study showing that 53% of patients with IBD will have opioid exposure within five years of diagnosis^34^ but further illustrate how these high rates of opioid prescription have persisted over time. The continued high rates of opioid use are in the context of increasing rates of musculoskeletal symptoms seen in our study, which are consistent with published global trends and may be related to increased provider awareness and an increase in the median age of the US population over time.^35^, ^36^ The increased specificity of musculoskeletal symptom codes in the transition from ICD‐9 to ICD‐10 in 2015 likely explains the significant increase in musculoskeletal symptom codes observed in that year,^37^ but there is still an overall increase in the rates of musculoskeletal codes across the study independent of the ICD transition period. Ultimately, the pervasive use of opioids in both adults and children, along with increasing rates of musculoskeletal pain, highlight the ongoing challenge to identify ways of providing effective, yet safe, analgesia in IBD across the age spectrum.

Several factors were found to be significantly associated with prescription NSAID use. Opioid prescription had the strongest independent association because patients receiving opioids are more likely to have pain and severe disease^15^, ^16^ and thus more likely to receive other analgesics. Inflammatory arthritis, osteoarthritis, other joint pain NOS, and chronic musculoskeletal pain were all associated with an increased odds of NSAID fill because NSAIDs are commonly prescribed for these indications. In particular, NSAIDs are considered first‐line therapy for several musculoskeletal conditions associated with IBD, such as enthesitis, axial spondyloarthritis, and psoriatic arthritis,^38^, ^39^ highlighting the relevance of this drug class in specific subpopulations of patients with IBD as a potentially safer alternative analgesic. Male sex was independently associated with a lower odds of NSAID prescription fill, possibly because of the greater burden of comorbidities in male patients that are relative contraindications to NSAID use (eg, CVD). Furthermore, women with IBD and spondyloarthritis have been shown to have poorer quality of life metrics and increased self‐reported pain compared to men, which may also influence NSAID prescription.^40^, ^41^ Age <18 years, as discussed previously, and age ≥80 years were associated with a lower odds of NSAID use. Elderly patients are more likely to have comorbidities that may preclude NSAID use and are known to have higher risks associated with exposure to this drug class.^42^ Interestingly, although several covariates in our study such as depression, anxiety, and substance use have been previously reported to be associated with opioid prescription,^12^, ^14^, ^15^, ^16^ they did not have particularly strong associations with NSAID use in our cohort.

There are several limitations to this study that should be acknowledged. Prescription fills may not always translate to actual patient use, which would bias toward overestimating NSAID use, although this overestimation is likely outweighed by an inability to assess over‐the‐counter NSAID use, leading to an underestimation of total NSAID use. However, this study is specifically evaluating patterns of prescription NSAID fills and how these patterns have changed over time. Furthermore, given recent guidelines making strong recommendations against NSAID use in IBD, patients likely are frequently counseled on avoiding over‐the‐counter NSAIDs. There is a potential of misclassification of patients as having IBD when using claims data, but validated algorithms were adapted using multiple IBD codes separated over time. Patients had to be enrolled for at least six months in a calendar year for data inclusion, but it is possible those enrolled for longer periods in a given year (ie, 12 months) would have a greater potential for NSAID exposure than those enrolled for a shorter period. The number of eligible months per calendar year was therefore adjusted for in our multivariable model. Although data were lacking on important factors that may serve as unmeasured confounders, such as IBD disease severity, codes for covariates that are important indications for NSAID prescription in IBD were included, namely arthritis and chronic musculoskeletal pain but without the ability to confirm such diagnoses when using claims data. Data on prescriber specialty and surgical codes were not assessed, and thus the association of these factors with prescription NSAID fills was not ascertained. There is a possibility of missing data if certain diagnoses were not coded for during insurance billing, but this issue is inherent to retrospective administrative data and missingness is expected to be nondifferential between NSAID exposure groups. Finally, although our study did not include uninsured individuals, our findings in this large national cohort are likely generalizable to other commercially insured patients with IBD in the US.

In conclusion, prescription NSAIDs are used in a minority of patients of all ages with IBD, but prescription fill rates have decreased over time, particularly for COX‐2 selective NSAIDs after 2004 and more generally after the recent ACG IBD treatment guidelines in 2018 and 2019. Pediatric patients were much less likely to receive prescription NSAIDs than adults, even though the side effect profile may be more favorable in this population. Although greater use of biologic/tsDMARDs may be partially responsible for lower rates of NSAIDs, the continued high rates of opioid use and coding for musculoskeletal complaints demonstrate an important remaining gap in pain treatment in this population. There is a need to better evaluate NSAID safety in both adults and children with IBD to determine whether NSAIDs can be an effective and potentially lower‐risk analgesic option than opioids for these patients.

## AUTHOR CONTRIBUTIONS

All authors contributed to at least one of the following manuscript preparation roles: conceptualization AND/OR methodology, software, investigation, formal analysis, data curation, visualization, and validation AND drafting or reviewing/editing the final draft. As corresponding author, Dr Mayer confirms that all authors have provided the final approval of the version to be published and takes responsibility for the affirmations regarding article submission (eg, not under consideration by another journal), the integrity of the data presented, and the statements regarding compliance with institutional review board/Declaration of Helsinki requirements.

## Supporting information


**Disclosure Form**:

**Data S1** Supporting Information
