# Supplementary material for: Temporal Trends in and Associations With Nonsteroidal Anti‐inflammatory Drug Prescription in Adult and Pediatric Patients With Inflammatory Bowel Disease
Source: Arthritis Care Res (Hoboken). 2025 Dec 17;78(4):439–48. doi: 10.1002/acr.25650 (PMC13034101; doi:10.1002/acr.25650)
Supplement: Supplementary file 2 — Data S1 Supporting Information [file ACR-78-439-s001.docx]

**DATA SUPPLEMENT**

**Supplementary Table 1.** Nonsteroidal anti-inflammatory drugs (NSAIDs) included in study

| Celecoxib | Indomethacin | Oxaprozin |
| --- | --- | --- |
| Diclofenac | Ketoprofen | Piroxicam |
| Diclofenac/misoprostol | Ketorolac | Rofecoxib |
| Diflunisal | Meclofenamate | Salsalate |
| Etodolac | Meloxicam | Sulindac |
| Fenoprofen | Nabumetone | Tolmentin |
| Flurbiprofen | Naproxen | Valdecoxib |
| Ibuprofen | Naproxen/sumatriptan |  |

**Supplementary Table 2. International Classification of Diseases (ICD)-9 and -10 diagnosis codes for covariates**

| Covariate | ICD-9 Codes | ICD-10 Codes |
| --- | --- | --- |
| Inflammatory arthritis (including rheumatoid arthritis, spondyloarthritis, and juvenile idiopathic arthritis) | 99.3, 696.0, 713.1, 714.xx other than 714.89-714.9x, 720.x | L40.5x, M02.3x, M05.xx, M06.0x, M06.2x, M06.3x, M06.8x, M06.9x, M07.xx, M08.xx, M45.xx, M46.1x, M46.8x-M46.9x |
| Osteoarthritis | 715.00, 715.04, 715.09-715.18, 715.20-715.28, 715.30-715.38, 715.80, 715.89-715.98, 721.0-721.3, 721.90, 721.91 | M15.xx-M19.xx |
| Other joint pain not otherwise specified (NOS) | 714.89, 714.9x | M06.4x, M12.0x-M12.1x, M12.3x, M12.8x-M12.9x, M13.0x-M13.1x, M13.8x, M14.8x, M25.4x-M25.6x, M25.8x-M25.9x, M79.6x |
| Chronic musculoskeletal pain | 307.80, 307.89, 338.0, 338.20-338.22, 338.28-338.29, 338.4x, 729.1x | F45.4x, G89.0, G89.2x, G89.4x |
| Depression | 293.83, 296.2x, 296.3x, 298.0x, 300.4, 309.0, 309.1, 309.28, 311 | F32.xx, F33.xx, F06.30, F06.31, F06.32, F34.9, F39, F43.21, F43.23 |
| Anxiety | 293.84, 300.2x, 300.0x, 308.9, 309.24, 309.89, 309.9 | F06.4, F40.xx, F41.xx, F43.0, F43.22, F43.8, F43.9 |
| Psychotic disorders | 296.0x, 296.1x, 296.4x, 296.5x, 296.6x, 296.7x, 296.8x, 296.9x, 295.xx, 297.xx, 298.xx, 780.1 | F30.xx, F31.xx, F06.33, F20.xx, F22, F23, F24, F25.xx, F28, F29, R44.xx |
| Substance use | 292.xx, 304.xx, 305.2x-305.9x, V65.42 | F11.xx, F12.xx, F13.xx, F14.xx, F15.xx, F16.xx, F18.xx, F19.xx, F55.xx, Z71.51 |
| Alcohol use | 291.xx, 303.xx, 305.0x, 571.0x-571.3x, E860.0, V11.3 | F10.xx, Z71.41, K70.xx |
| Nicotine/tobacco use | 305.1, 989.84, V15.82 | F17.xx, T65.2xx, Z87.891 |

**Supplementary Figure 1.** Longitudinal trends in the most commonly-filled nonselective and COX-2 selective NSAIDs


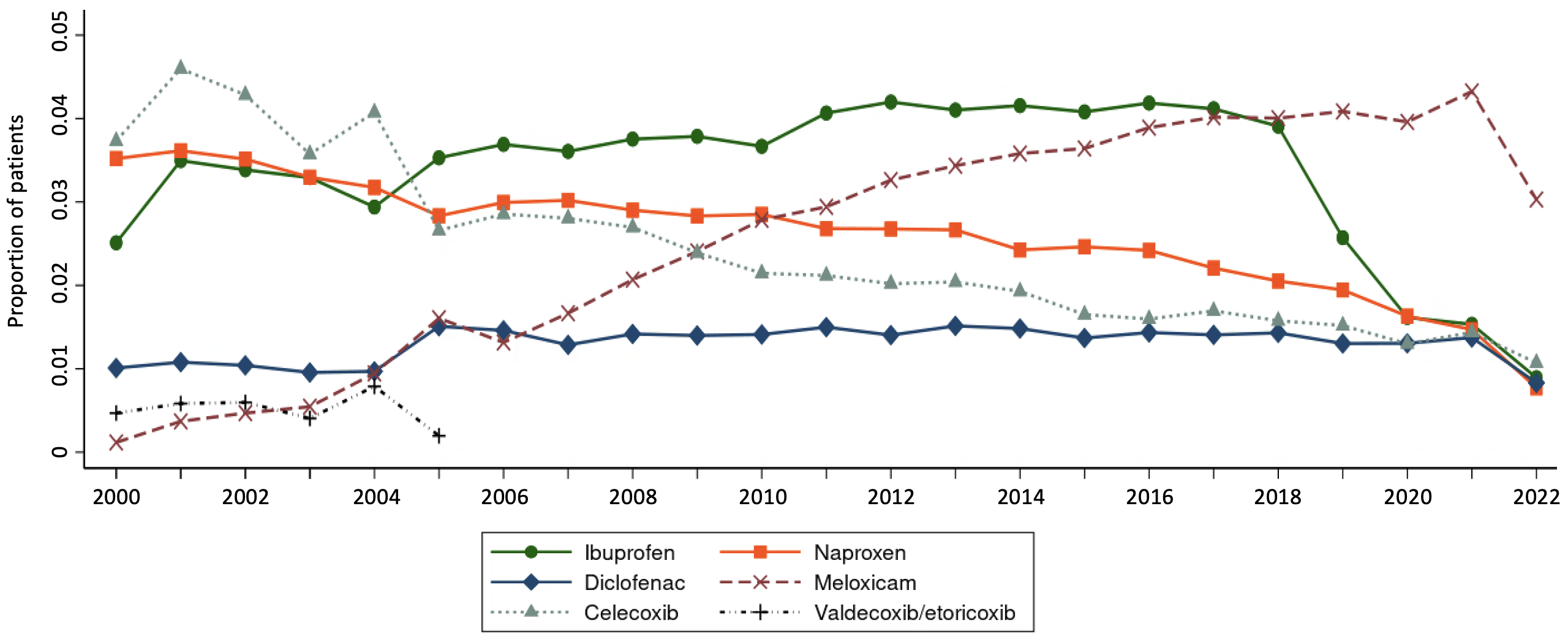


*Legend: COX: cyclo-oxygenase, NSAID: nonsteroidal anti-inflammatory drug. All COX-2 selective NSAIDs and nonselective NSAIDs with fills in >1% of observations included.*

**Supplementary Figure 2.** Longitudinal NSAID (panel A) and opioid (B) fill trends across IBD subtypes

**
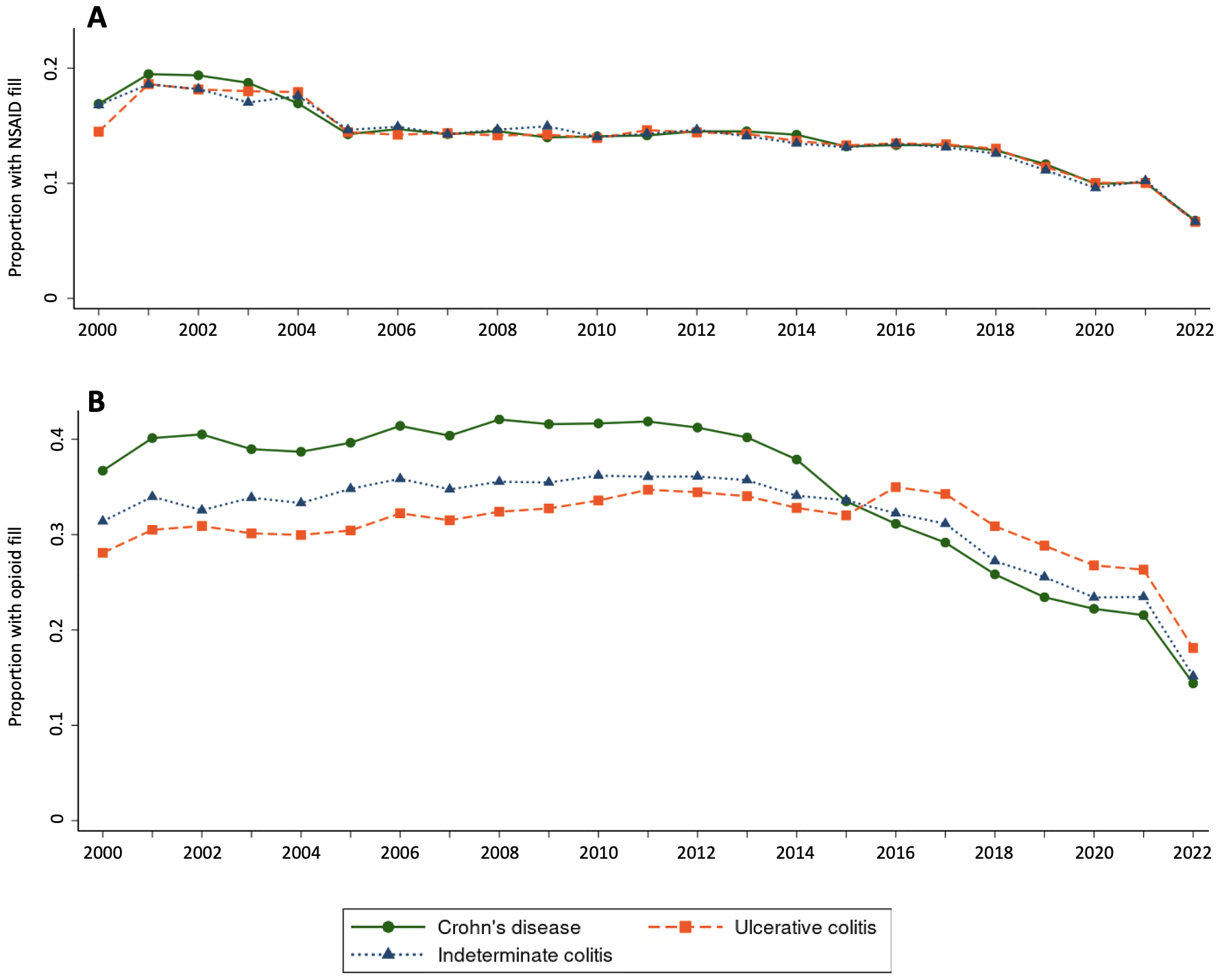
**

*Legend: NSAID: nonsteroidal anti-inflammatory drug; IBD: inflammatory bowel disease*

**Supplemental Figure 3.** Longitudinal NSAID and opioid fill trends amongst chronic users

**
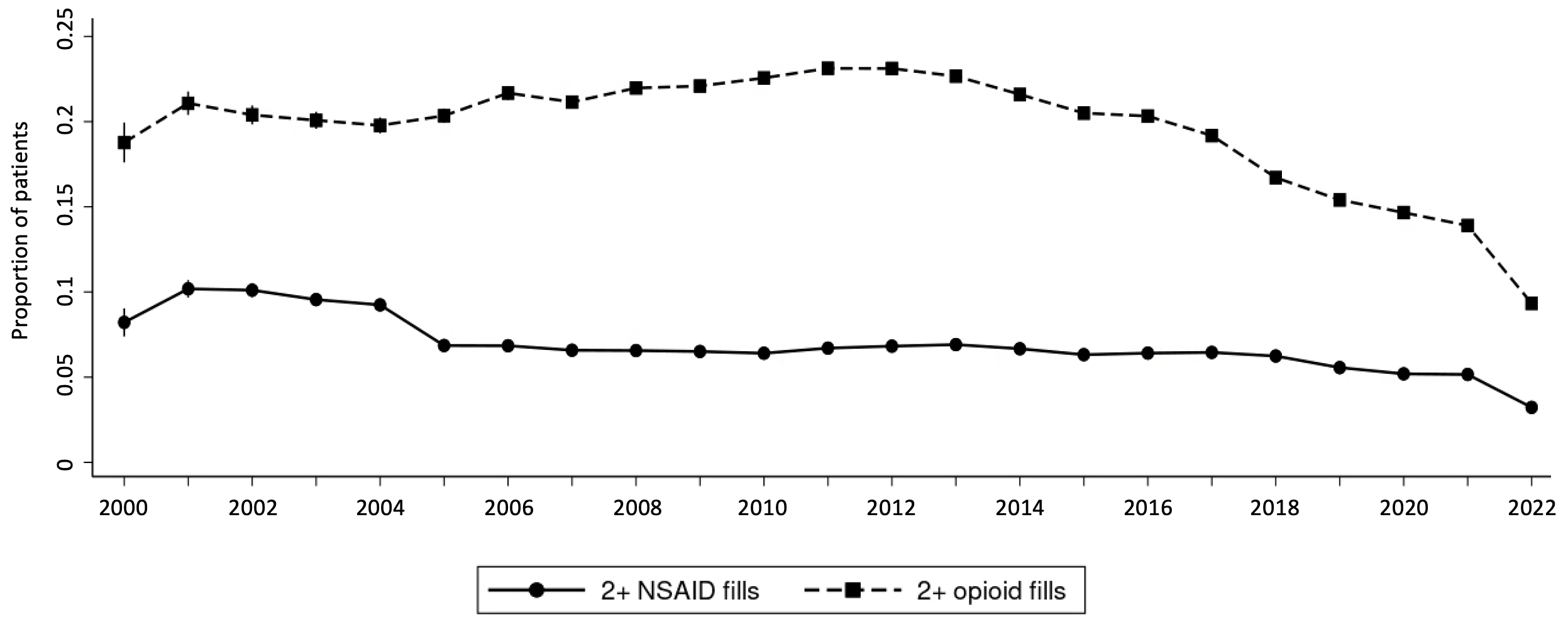
**

*Legend: NSAID: nonsteroidal anti-inflammatory drug; chronic users defined as* $\geq$*2 fills within a calendar year*
